# Inflammation and nerve injury induce expression of pancreatitis-associated protein-II in primary sensory neurons

**DOI:** 10.1186/1744-8069-6-23

**Published:** 2010-04-26

**Authors:** Shao-Qiu He, Jun-Ru Yao, Fang-Xiong Zhang, Qiong Wang, Lan Bao, Xu Zhang

**Affiliations:** 1 Institute of Neuroscience and State Key Laboratory of Neuroscience, Shanghai Institutes for Biological Sciences, Chinese Academy of Sciences, 320 Yue Yang Road, Shanghai 200031, China; 2 Laboratory of Molecular Cell Biology, Institute of Biochemistry and Cell Biology, Shanghai Institutes for Biological Sciences, Chinese Academy of Sciences, Shanghai 200031, China

## Abstract

Pancreatitis-associated protein (PAP)-I and -II, lectin-related secretory proteins, are members of the regenerating gene (Reg) family. Although expression of PAP-I was found in the dorsal root ganglion (DRG) neurons following peripheral nerve injury and cystitis, whether PAP-II could be expressed in DRG neurons in chronic pain models remains unclear. The present study shows an inflammation- and nerve injury-triggered expression of PAP-II in rat DRG neurons. *In situ *hybridization showed that only a few DRG neurons normally contained PAP-I and -II mRNAs. After peripheral inflammation, PAP-I and -II mRNAs were present in over half of small DRG neurons. Such an elevated expression of PAP-I and -II reached the peak level on the second day. Immunostaining showed that the expression of PAP-II was mostly increased in the isolectin B4-positive subset of small DRG neurons after inflammation. Furthermore, the expression of PAP-II was also induced in DRG neurons after peripheral nerve injury. Interestingly, PAP-II expression was shifted from small neurons on day 2 to large DRG neurons that expressed neuropeptide Y during the later post-injury days. These results suggest that PAP-II may play potential roles in the modulation of spinal sensory pathways in pathological pain states.

## Background

Tissue inflammation or lesions to the nervous system may result in enhanced responses to noxious stimuli or pain evoked by normally innocuous stimuli [1-6]. Accumulated evidence has shown that many neurotransmitters/neuromodulators, receptors, ion channels and signaling molecules are involved in the generation of chronic pain [1,2,6,7]. Nociceptors are known to be sensitized by a number of inflammatory mediators released from damaged tissues, such as ATP, nitric oxide, interleukin 1, interleukin 6, and tumour necrosis factor alpha (TNF-α) [1,2,6]. Moreover, changes in the gene expression profiles of the dorsal root ganglion (DRG) and that of the dorsal spinal cord following peripheral tissue inflammation or peripheral nerve injury may contribute to the mechanisms for the initiation and development of pathological pain [4,6,8]. Therefore, it is interesting to identify the molecules that are strongly regulated in the spinal sensory pathways following peripheral tissue inflammation or nerve injury.

Pancreatitis-associated protein-I (PAP-I) is known as PAP 1 and peptide 23 in the rat, and regenerating (Reg) islet-derived protein 3 beta (Reg III-β) in the mouse, and PAP-II known as Reg III in the rat and Reg III-α in the mouse. They are lectin-related secretory proteins and belong to the Reg family which contains similar proteins found in the pancreas and gastrointestinal tract in physiological and/or pathological conditions [9-11]. *Reg *is first isolated from a regenerating islet cDNA library [12] and encodes a secretory protein with a growth stimulating effect on pancreatic β cells [10,13]. Based on the primary structures of the proteins, the members of the Reg family are grouped into three subclasses, namely type I, II and III [10,14-18]. PAP-I and -II are members of the type III subclass and encoded by gene PAP-I and PAP-II, respectively.

Previous studies suggest that both PAP-I and -II are involved in the modulation of inflammatory responses. During acute pancreatitis, PAP-I may contribute to stress response to control bacterial proliferation [9]. In pancreatic AR4-2J cells, PAP-I is one of the effectors for the TNF-α-induced apoptosis inhibition [19]. Although PAP-II is first isolated as a pancreatic secretory protein that contributes to pancreatic regeneration, it is up-regulated during the acute phase of pancreatitis and likely modulates the inflammatory environment of pancreatitis [9,20-24]. PAP-II is found to inhibit TNF-α-mediated inflammatory responses [25,26]. In the exocrine pancreas, PAP-I is associated with pancreatic acinar cell and protects cells from oxidative stress and TNF-α-induced pancreatic stress [19,27]. Moreover, expression of PAP-II is also increased in gut epithelial cells in human inflammatory bowel disease [28].

Several reports suggest that Reg proteins could also be functional in the nervous system. The Reg-1, a member of the Reg type I subclass, is expressed in the brain during development and Alzheimer's disease [13,29]. PAP-I may contribute to the signaling pathway of ciliary neurotrophic factor and is involved in the regeneration and survival of motor neurons [30,31]. The expression of PAP-I has been found to be induced in urinary tract afferent neurons following cyclophosphamide-induced cystitis [32], suggesting its potential role in the abnormal sensation in cystitis. Moreover, the PAP-I expression in isolectin B4 (IB4)-positive small DRG neurons is up-regulated and followed by a dynamic shift from small to large DRG neurons after peripheral nerve injury [33]. Increased expression of PAP-I also occurs in neurons following traumatic brain injury [34]. These data indicate that PAP-I expression in response to injury and inflammation could be a general response in the pancreas, intestine, and both peripheral and central nervous systems. However, it remains unclear whether PAP-II is involved in the response of primary sensory neurons to the stimulations of peripheral inflammation and nerve injury. The present study shows that the expression of PAP-II is strongly induced in DRG neurons following peripheral tissue inflammation and nerve injury, suggesting an involvement of PAP-II in the signal processing of the spinal sensory pathways in chronic pain states.

## Results

### Peripheral inflammation-triggered expression of PAPs in small DRG neurons

Increase in expression of PAP-I and -II in the rat DRG after peripheral tissue inflammation was suggested by our gene microarray analysis. We found that their expression was markedly increased in lumbar (L) 4 and L5 DRGs when peripheral tissue inflammation was induced by intraplantar CFA injection into the hindpaw of rats. The microarray signal for PAP-I mRNA was increased by 2394%, 1660% and 1128% at day 2, 4 and 7 after CFA injection, respectively, while the signal for PAP-II mRNA was increased by 87% at day 2, 134% at day 4 and 94% at day 7. These results suggest an inflammation-induced expression of PAP-I and -II in the DRG.

**Figure 1:**
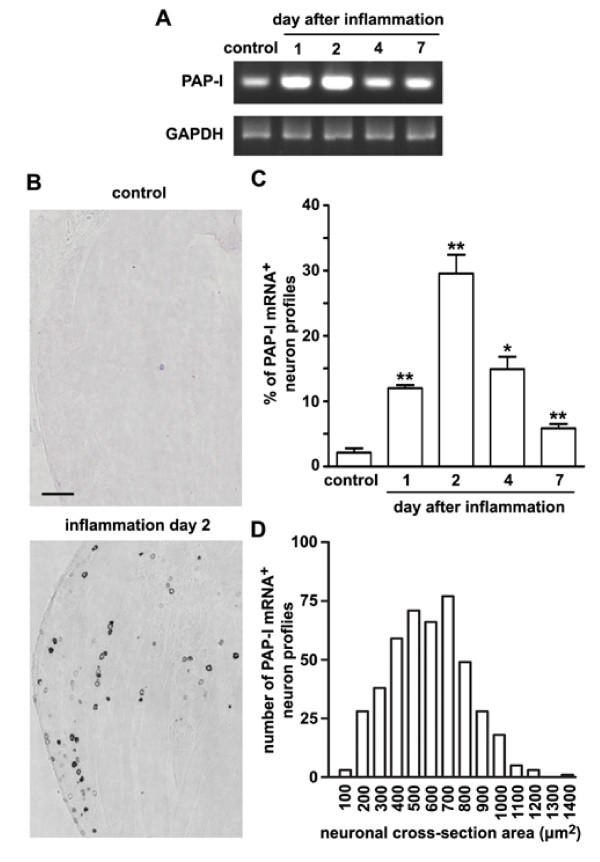
**Up-regulation of PAP-I in rat DRGs after peripheral inflammation**. (A) RT-PCR shows the increases in PAP-I mRNA levels in L4 and L5 DRGs after intraplantar CFA injection. (B) *In situ *hybridization shows that a few small neurons contain PAP-I mRNA in the DRG of normal rats. Two days after intraplantar CFA injection, the number of PAP-I mRNA-containing DRG neurons is markedly increased. Scale bar = 200 μm. (C) Time course of the up-regulated expression of PAP-I following CFA-induced inflammation shows that the increase in the number of PAP-I mRNA-containing DRG neurons reaches the peak level 2 days after inflammation and, then, is gradually reduced. Quantification was performed on 2~3 sections for each DRG from at least three animals at each time point. * *P *< 0.05, ** *P *< 0.01 versus control. (D) The PAP-I mRNA-containing neurons are mostly small DRG neurons (neuronal cross-section area < 900 μm^2^, n = 446).

We then used RT-PCR to confirm the expression of PAP-I and -II in the rat DRG following peripheral inflammation. The level of PAP-I mRNA was markedly elevated in the DRG one day after inflammation (Fig. 1A). Such an increased expression reached the peak level at day 2 and remained at a reduced level, which was still higher than control, during 4-7 days after inflammation (Fig. 1A). *In situ *hybridization experiments showed that only ~2% of total neurons in control L4 and L5 DRGs contained PAP-I mRNA (Fig. 1B and 1C). The number of PAP-I mRNA-containing neurons was markedly increased to ~28% of total neurons at inflammation day 2 (Fig. 1B and 1C). Analysis of cell-size distribution showed that most of these neurons were small DRG neurons (Fig. 1B). Quantitative analysis showed that the PAP-I mRNA-containing neuron profiles had a cross-section area ranging from 100 to 1400 μm^2 ^with a peak area between 400 to 700 μm^2 ^(Fig. 1D). There were still ~6% of DRG neurons containing PAP-I mRNA at day 7 (Fig. 1C). These results indicate that the expression of PAP-I mRNA is induced in small DRG neurons after peripheral inflammation.

**Figure 2:**
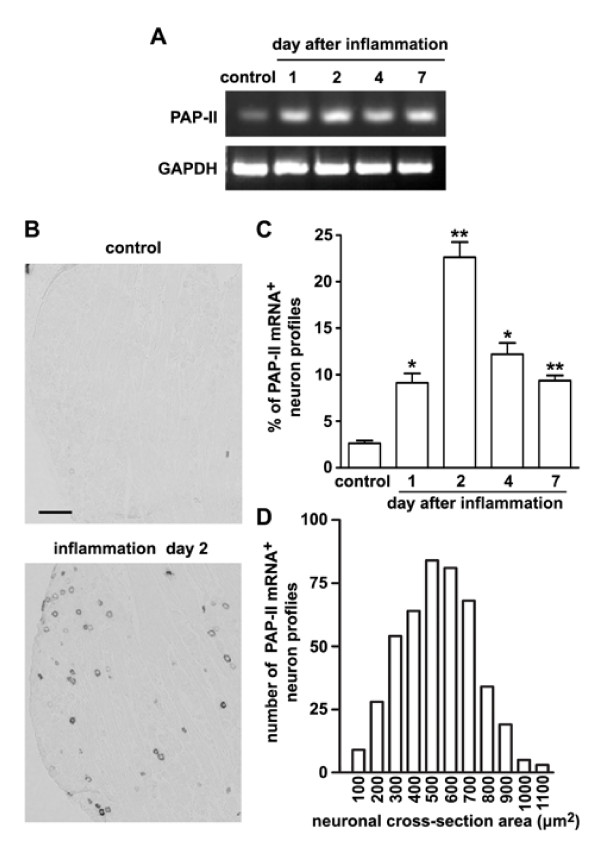
**Expression of PAP-II in small DRG neurons after peripheral inflammation**. (A) RT-PCR shows the increased level of PAP-II mRNA in L4 and L5 DRGs after intraplantar CFA injection. (B) *In situ *hybridization shows that PAP-II mRNA is not found in DRG neurons of the normal rat. Two days after CFA-induced inflammation, PAP-II mRNA is present in many DRG neurons. Scale bar = 200 μm. (C) The increase in number of PAP-II mRNA-containing DRG neurons reaches the peak level 2 days after CFA injection, and remains at a lower level 7 days after inflammation. Quantification is performed on 2~3 sections for each DRG from at least three animals at each time point. * *P *< 0.05, ** *P *< 0.01 versus control. (D) These PAP-II mRNA-containing neurons are mostly small DRG neurons (neuronal cross-section area < 900 μm^2^, n = 450).

Similar to PAP-I, only a low level of PAP-II mRNA was found in L4 and L5 DRGs of normal rats. However, the expression level of PAP-II was markedly increased in the DRGs one day after intraplantar CFA injection (Fig. 2A). Such an elevated expression reached the peak level at day 2, and lasted at least for 7 days (Fig. 2A). *In situ *hybridization histochemistry was then employed to illustrate the cellular distribution of PAP-II mRNA. We found that less than ~3% of total neurons contained PAP-II mRNA in the DRG of control group (Fig. 2B and 2C). However, the percentage of PAP-II mRNA-containing DRG neurons was markedly increased, reaching ~23% of total neurons 2 days after inflammation (Fig. 2B and 2C). Most of these neurons are small DRG neurons (Fig. 2B and 2D). Seven days after peripheral inflammation, there were still ~10% of DRG neurons containing PAP-II mRNA (Fig. 2C). These results indicate that peripheral tissue inflammation triggers the expression of PAP-II mRNA in nociceptive small DRG neurons.

### Presence of PAP-II in IB4-positive subset of small DRG neurons after inflammation

**Figure 3:**
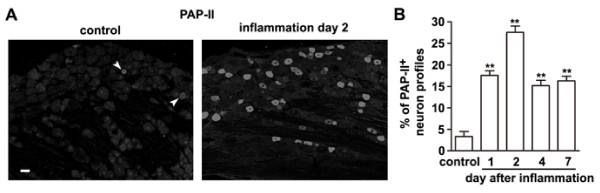
**Increase in PAP-II immunostaining in rat DRGs after peripheral inflammation**. (A) Immunohistochemistry shows that the number of small DRG neurons immunostained for PAP-II is apparently increased 2 days after CFA injection. (B) The percentage of PAP-II-immunostained small DRG neurons is significantly increased from 3.3 ± 1.2% in the control DRGs to 27.6 ± 1.5% in the DRGs 2 days after peripheral inflammation. Such an increase of PAP-II-positive DRG neurons is reduced 4 days after inflammation, but remains at the low level 7 days after inflammation. Scale bar = 50 μm. ** *P *< 0.01 versus control (n = 7 rats).

We next asked whether the increase in PAP-II mRNA resulted in an elevated protein level in small DRG neurons. Immunostaining showed that only ~3% of DRG neurons of normal rats contained PAP-II (Fig. 3A and 3B), consistent with the result of *in situ *hybridization. However, the number of PAP-II-immunoreactive neurons was markedly increased in L4 and L5 DRGs after peripheral inflammation induced by intraplantar CFA injection (Fig. 3A and 3B). Quantitative analysis showed that ~28% of total DRG neurons in the DRG at inflammation day 2 contained PAP-II (Fig. 3B). Most of PAP-II-immunoreactive neuron profiles (n = 220) were small DRG neurons which had a cross-section area less than 900 μm^2^, and covered 45% of all small neurons. Although the number of PAP-II-positive neurons was decreased to ~15% of total neurons at day 4, PAP-II was still present in many DRG neurons (~16%) at day 7, suggesting a long-lasting up-regulation of this protein. Thus, peripheral inflammation induces the expression of PAP-II in small DRG neurons.

**Figure 4:**
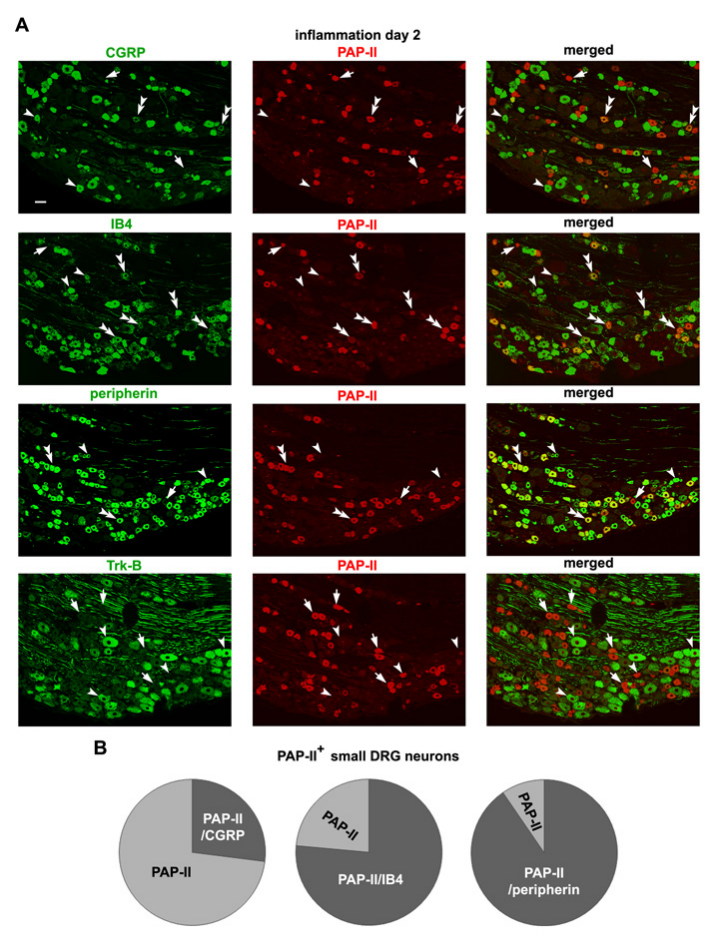
**Distribution of PAP-II in subsets of small DRG neurons 2 days after inflammation**. (A) Double-immunofluorescence staining shows that in the rat DRG the majority of PAP-II-positive small DRG neurons do not contain CGRP, but are positive for IB4 2 days after CFA injection. Most PAP-II-containing DRG neurons are immunostained for peripherin. There is no co-localization of PAP-II and Trk-B in DRG neurons. PAP-II-positive neurons co-expressing other molecules, PAP-II-negative neurons but containing other molecules and PAP-II-positive neurons negative for other molecules are indicated by double arrowheads, single arrowhead and arrow, respectively. Scale bar = 50 μm. (B) Quantitative analysis shows that 26.3 ± 3% of PAP-II-positive DRG neurons (n = 352) co-express CGRP 2 days after peripheral inflammation, and 76.7 ± 1.4% (n = 399) stained for IB4. Most PAP-II-containing DRG neurons (89.8 ± 3.3%; n = 293) are immunostained for peripherin.

We further identified the subset of small DRG neurons that expressed PAP-II following peripheral inflammation. Small DRG neurons can be divided roughly into two subsets, namely the peptidergic subset containing neuropeptide substance P and calcitonin gene-related peptide (CGRP) and non-peptidergic subset positive for IB4, although there is considerable overlap between these two subsets of small neurons. Double-immunofluorescence staining showed that two days after peripheral tissue inflammation, most of PAP-II-immunoreactive small DRG neurons (76.7 ± 1.4%) were also IB4-positive, while 26.3 ± 3.0% of PAP-II-positive neurons contained CGRP (Fig. 4A and 4B). About 89% of PAP-II-positive DRG neurons were also immunostained for peripherin (Fig. 4A and 4B), a marker for small DRG neuron [35-37]. In contrast, PAP-II was absent in Trk-B-immunoreactive DRG neurons (Fig. 4A), suggesting that inflammation-induced expression of PAP-II mainly occurs in small DRG neurons. Thus, the expression of PAP-II is induced mainly in IB4-positive subset of small DRG neurons.

### Peripheral nerve injury-induced expression of PAP-II in DRG neurons

**Figure 5:**
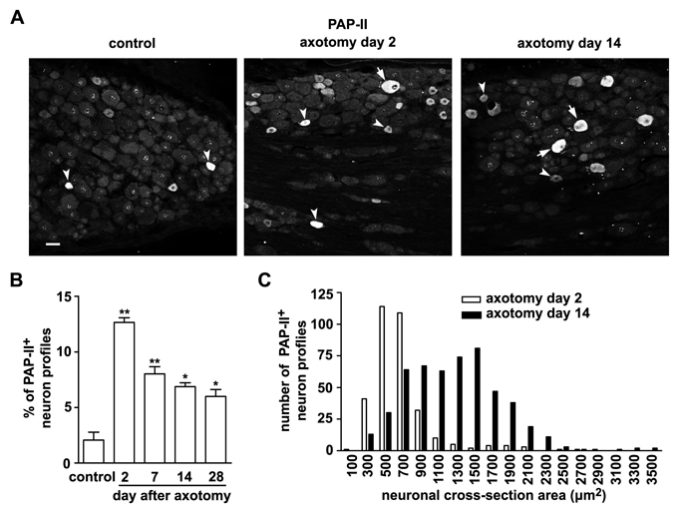
**Increase in PAP-II-immunostained DRG neurons after peripheral nerve injury**. (A) Immunohistochemistry shows that the number of PAP-II-containing DRG neurons is markedly increased in the rat DRG after sciatic nerve axotomy. Single arrowhead and arrow indicate PAP-II-positive small or large DRG neurons, respectively. Scale bar = 50 μm. (B) The percentage of PAP-II-immunostained DRG neurons is significantly increased from 2.1 ± 0.7% in the DRGs of normal rats to 12.7 ± 0.4% and 6.9 ± 0.6% in the DRGs 2 and 14 days after peripheral axotomy, respectively. * *P *< 0.05, ** *P *< 0.01 versus control (n = 3 rats). (C) Two days after sciatic nerve transection, PAP-II-immunoreactivity is mainly present in small neurons (neuronal cross-section area < 900 μm^2^) and a few large neurons (> 900 μm^2^) (n = 332). Fourteen days after axotomy, the number of PAP-II-positive large DRG neurons (n = 514) is markedly increased, whereas the number of PAP-II-containing small neurons is decreased.

It has been reported that the PAP-I expression in IB4-positive small DRG neurons is up-regulated and followed by a shift from small to large DRG neurons after peripheral nerve injury [33]. Therefore, we are interested to know whether peripheral nerve injury could induce the expression of PAP-II in DRG neurons. We found that the expression of PAP-II was also induced in DRG neurons after peripheral nerve injury. Immunostaining showed that only ~2% of DRG neurons were positive for PAP-II in L4 and L5 DRGs on the contralateral side after unilateral axotomy of the sciatic nerve (Fig. 5A and 5B), similar to that found in the DRGs of normal rats. However, the number of PAP-II-immunoreactive neurons was markedly increased in the ipsilateral L4 and L5 DRGs (Fig. 5A and 5B). Quantitative analysis showed that ~13% of total DRG neurons contained PAP-II in the ipsilateral DRGs 2 days after unilateral axotomy (Fig. 5B). Most of PAP-II-immunoreactive DRG neurons were small ones at this time interval (Fig. 5A and 5C).

Fourteen days after unilateral axotomy of the sciatic nerve, PAP-II was still present in many DRG neurons (~7% of total neurons), indicating a long-lasting up-regulation of this protein (Fig. 5A and 5B). However, there was a dynamic shift from small DRG neurons to large ones (Fig. 5A). In contrast to the presence of PAP-II-immunoreactivity in small DRG neurons 2 days after axotomy, the majority of PAP-II-positive DRG neurons was large ones (> 900 μm^2^) 14 days after axotomy (Fig. 5C). Therefore, the expression of PAP-II is rapidly up-regulated in many small DRG neurons 2 days after peripheral axotomy, followed by reduction in small DRG neurons but further up-regulation in large neurons.

### Distribution of PAP-II in neuropeptide Y-containing large DRG neurons after peripheral nerve injury

**Figure 6:**
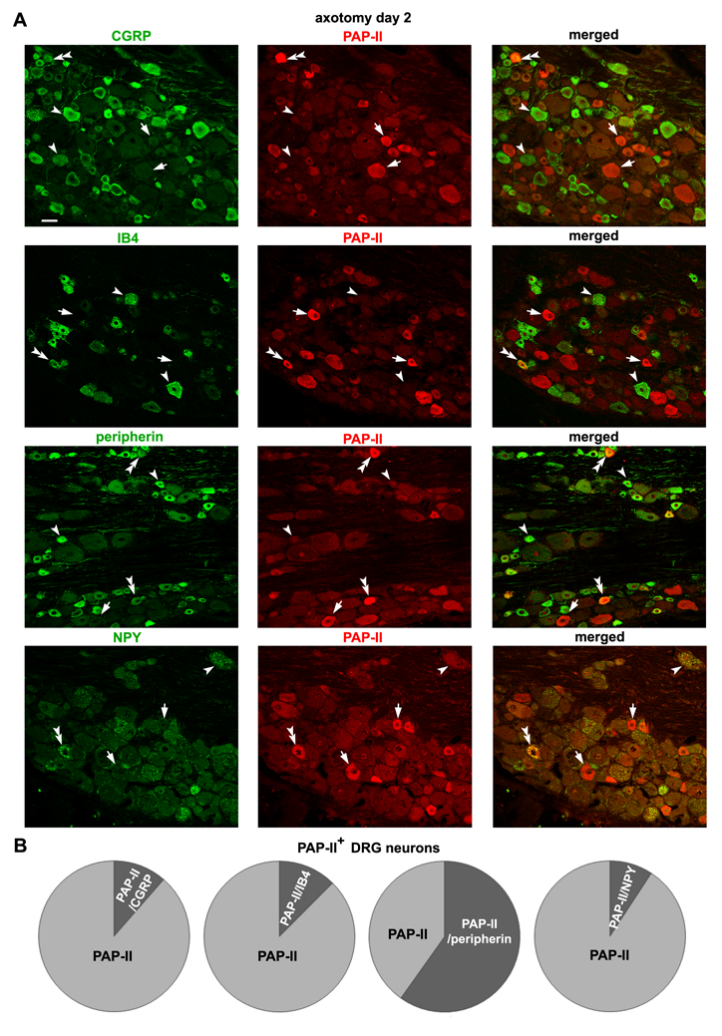
**Distribution of PAP-II in DRG neurons 2 days after nerve injury**. (A) Double-immunofluorescence staining shows that the majority of PAP-II-positive small DRG neurons are IB4-, CGRP- and NPY-negative 2 days after sciatic nerve axotomy. However, ~60% of the PAP-II-containing DRG neurons are peripherin-immunoreactive. Double arrowheads, single arrowhead and arrow indicate PAP-II-positive neurons co-expressing other molecules, PAP-II-negative neurons but containing other molecules, and PAP-II-positive neurons absent for other molecules, respectively. Scale bar = 50 μm. (B) Quantitative analysis shows that 10.9 ± 1.6% PAP-II-immunoreactive DRG neurons (n = 271) also express CGRP 2 days after nerve axotomy, while 12.6 ± 2.0% (n = 217) and 8.9% ± 1.7% (n = 379) PAP-II-positive DRG neurons are stained for IB4 or NPY respectively. More than half of the PAP-II-containing DRG neurons (54.6 ± 10.3%, n = 254) are peripherin-immunoreactive neurons.

Double-immunofluorescence staining showed that 2 days after peripheral nerve injury, most of PAP-II-immunoreactive small DRG neurons were also IB4 or CGRP-negative (Fig. 6A and 6B), while ~60% of PAP-II-positive DRG neurons were immunostained for peripherin (Fig. 6A and 6B). It is known that neuropeptide Y (NPY) is expressed at very low levels in DRG neurons of normal rats, but its expression is markedly up-regulated in large DRG neurons after peripheral nerve injury [38,39]. Although the expression of NPY in DRG neurons remained at low levels 2 days after sciatic nerve axotomy, the expression of PAP-II had already been up-regulated in DRG neurons including some large neurons (Fig. 6A). Therefore, less than 10% of PAP-II-positive neurons contained NPY (Fig. 6A), suggesting that nerve injury-induced expression of PAP-II may represent an early response of DRG neurons to injury.

**Figure 7:**
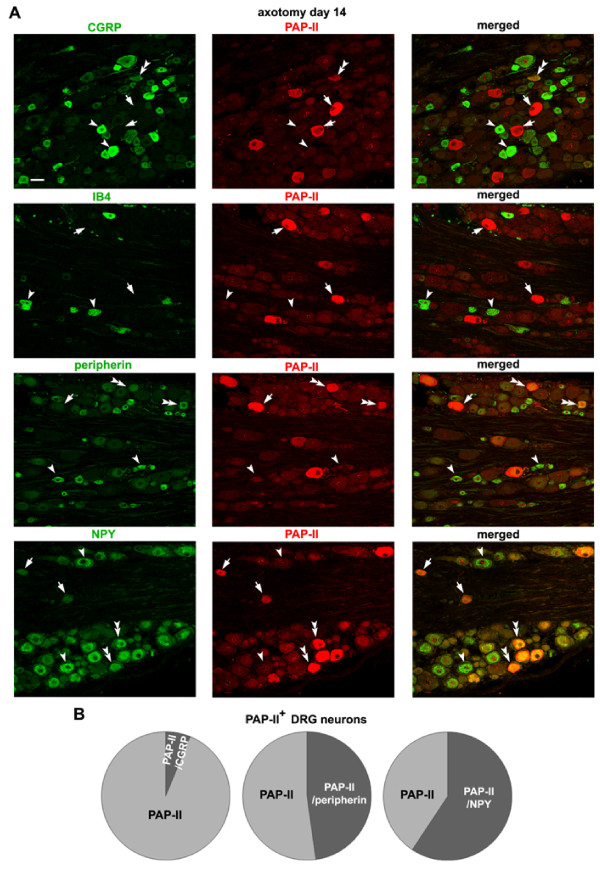
**Distribution of PAP-II in DRG neurons 14 days after nerve injury**. (A) Double-immunofluorescence staining shows that most of PAP-II-positive small DRG neurons do not contain CGRP and are also negative for IB4 14 days after sciatic nerve axotomy. However, ~50% of the PAP-II-positive DRG neurons contain peripherin. More than half of PAP-II-positive neurons contain NPY, and most of these neurons are large DRG neurons. Double arrowheads, single arrowhead and arrow indicate PAP-II-positive neurons co-expressing other molecules, PAP-II-negative neurons but containing other molecules, and PAP-II-positive neurons without other molecules, respectively. Scale bar = 50 μm. (B) Quantitative analysis shows that 6.3 ± 3.2% (n = 97) PAP-II-positive DRG neurons are stained for CGRP 14 days after axotomy, but none of PAP-II-positive DRG neurons (n = 120) are positive for IB4. About half of the PAP-II-immunoreactive DRG neurons (48.7 ± 8.7%, n = 136) are peripherin-positive, while 61.1 ± 2.8% (n = 177) of PAP-II-positive DRG neurons contain NPY.

The down-regulated expression of CGRP and IB4 in small DRG neurons, and the up-regulated expression of NPY in large DRG neurons became predominant 14 days after sciatic nerve axotomy (Fig. 7A), consistent with previous studies [38,39]. We found that only a few PAP-II-containing DRG neurons were immunostained for CGRP but none for IB4 (Fig. 7A and 7B). Although the percentage of PAP-II-positive neurons expressing peripherin was slightly decreased (from 54.6 ± 10.3% to 48.7 ± 8.7%), co-expression of PAP-II and peripherin was still present in small DRG neurons (Fig. 7A and 7B). The number of large DRG neurons that co-expressed PAP-II and NPY was markedly increased, contributing to 61.1 ± 2.8% of total PAP-II-containing DRG neurons (Fig. 7A and 7B). Thus, the expression of PAP-II can be induced in large DRG neurons that express NPY 14 days after peripheral nerve injury.

### Anterograde transport of PAP-II in sensory afferent fibers

**Figure 8:**
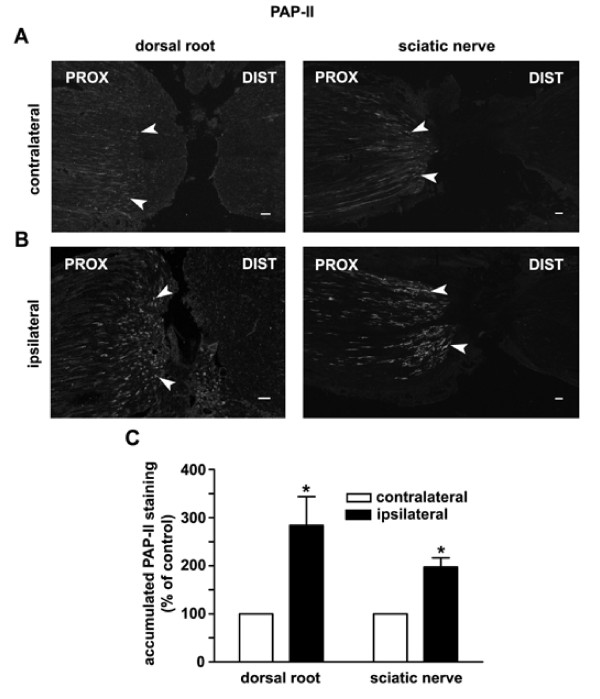
**Anterograde transport of PAP-II in afferent fibers**. (A, B) Immunostaining shows the presence of PAP-II in nerve fibers in the rat sciatic nerve and dorsal root that are proximal (PROX, arrowheads) to the DRG 24 h after nerve ligation, but absence in the distal (DIST) portion. The number of PAP-II-immunoreactive afferent fibers is increased in the proximal portion of ligated sciatic nerve and dorsal root from the rat that had undergone inflammation, whereas there is no obvious accumulation of the protein in the distal side. Scale bar = 50 μm. (C) Statistical analysis shows that the accumulation of the PAP-II in the proximal side of dorsal root is increased by ~200%, and that in the sciatic nerve is increased by ~100% (the immunofluorescence intensity are measured in the dorsal root and sciatic nerve 500 μm from ligation site). * *P *< 0.05 versus contralateral side (n = 5 rats).

Although PAP-II-immunoreactivity was found in the cell bodies of small DRG neurons, we failed to detect its presence in sensory afferent fibers in lamina I-II of the rat spinal cord. This might be due to either a lack of PAP-II transport in axons or a rapid release of PAP-II at afferent terminals. To test these possibilities, we examined whether PAP-II protein could be transported to nerve terminals. The sciatic nerve and dorsal root of rats were ligated for 1 day to attenuate the axonal transport of proteins, resulting in an accumulation of transported protein near the ligation site. Using immunostaining, we found that PAP-II was accumulated in the nerve fibers in the portion of the ligated sciatic nerve and dorsal root that was proximal to L4 and L5 DRGs but not in the distal portion (Fig. 8A and 8B), suggesting that PAP-II can be anterogradely transported to the afferent terminals. After peripheral tissue inflammation, the accumulation of PAP-II in the proximal side of the dorsal root and the sciatic nerve was significantly increased (Fig. 8B and 8C). As a control, an accumulation of CGRP was also found in the proximal portion of the ligated sciatic nerve and dorsal root (data not shown). These results suggest that newly synthesized PAP-II can be transported to sensory afferent terminals in the spinal cord and peripheral tissues.

## Discussion

The present study shows that expression of PAP-II in small DRG neurons can be induced by peripheral tissue inflammation and nerve injury. Peripheral tissue inflammation causes up-regulated expression of several neuropeptides which are normally expressed in DRG neurons, such as substance P and CGRP [40,41], consistent with their functional roles in the inflammatory responses [42,43]. However, PAP-II represents the secretory protein that is largely absent in DRG neurons under normal circumstance, but is expressed in these sensory neurons in response to the inflammatory stimulation and peripheral nerve injury. These results suggest a potential role of PAP-II in the modulation of the activity of spinal sensory circuits in pathological conditions.

In response to peripheral inflammatory stimuli, substance P and CGRP can be released from peptidergic afferent terminals to facilitate both peripheral inflammatory responses and nociceptive afferent synaptic transmission in the dorsal spinal cord [40,42,43], indicating a key role of peptidergic small DRG neurons in inflammatory pain. In the present study, we reveal that the inflammation-induced expression of PAP-II mainly occurs in IB4-positive subset of small DRG neurons but only in some peptidergic small DRG neurons, suggesting that PAP-II may be specifically involved in the inflammatory response of IB4-positve small DRG neurons. Moreover, Takahara et al. [32] reported that the expression of PAP-I was up-regulated in both IB4-positive and TrkA-positive (peptidergic) small DRG neurons following cyclophosphamide-induced cystitis. Therefore, both PAP-I and -II may contribute to the processing of inflammatory signals in nociceptive afferent neurons. Interestingly, the inflammation-induced PAP-II expression in IB4-positive subset of small DRG neurons suggests that this subset of sensory neurons may play an active role in the inflammatory response through a relatively selective mechanism, which might be different from that in peptidergic small DRG neurons.

In the present study, we failed to show PAP-II-immunoreactivity in afferent C- and Aδ-fibers in the superficial dorsal horn of the spinal cord. However, accumulated PAP-II was found in the proximal portion of ligated sciatic nerve and dorsal root, suggesting an existence of anterograde transport of this protein to nerve terminals. Therefore, the absence of PAP-II in afferent fibers might be due to the constitutive release of the protein. The PAP-II inhibits the TNF-α-induced inflammatory response in macrophages [25] and in epithelial and endothelial cells [26]. Interestingly, TNF-α is one of pro-inflammatory mediators involved in the sensitization of nociceptors [44-47]. TNF-α can be anterogradely transported from the DRG to the sciatic nerve [48] and the spinal cord [49]. This inflammatory mediator acts directly on nociceptive terminals that express TNF-α receptors [50,51]. Our present results show the up-regulated expression of PAP-II in nociceptive DRG neurons and the anterograde transport of PAP-II in sensory afferent fibers, suggesting that the PAP-II secreted from sensory afferents might regulate the pro-inflammatory response of TNF-α in both peripheral tissues and the dorsal spinal cord. This may represent a mechanism for the homeostatic regulation in the inflammation.

We find here that peripheral inflammation induces expression of both PAP-I and -II in small DRG neurons. Moreover, these proteins are known to exert the anti-inflammatory effects in pancreatic-injury [25] and inhibitory effects in TNF-α-induced inflammatory response [25,26]. Therefore, PAP-I and -II may be involved in both the inflammatory reaction and the regeneration process of damaged cells and tissues. Peripheral nerve injury leads to an up-regulated expression of PAP-I in IB4-positive small DRG neurons followed by a dynamic shift from small to large DRG neurons [33]. The present study shows that peripheral nerve injury induces PAP-II expression in peripherin-containing small DRG neurons which mostly expressed neither CGRP nor IB4, followed by a shift to NPY-containing large DRG neurons 14 days after peripheral axotomy. This nerve injury-induced distribution pattern of PAP-II in DRGs is distinct from that induced by peripheral inflammation, suggesting that the expression of PAP-II could be regulated through different mechanisms in pathological conditions.

Interestingly, up-regulation of PAP-II occurs in primary sensory neurons following both peripheral tissue inflammation and nerve injury that often cause chronic pain. In addition to the potential roles of PAPs in pain modulation, these proteins may play a role in nerve regeneration because their expression is markedly up-regulated after peripheral axotomy, which also initiates the process for nerve repair. Moreover, the findings of the PAP expression in IB4-positive neurons following peripheral inflammation and the shift of PAP expression between different subpopulations of DRG neurons after nerve injury suggest that different populations of DRG neurons may response to the inflammation and the nerve/tissue damage through relatively selective mechanisms. Therefore, it would be interesting to further investigate the functional roles of PAP-II in pathological pain and the related mechanisms. Since the patterns of up-regulated expression and cell distribution of PAP-I and PAP-II in the DRGs appear to be very similar, it would be necessary to know whether there is any functional difference between these proteins.

We conclude that both peripheral inflammation and nerve injury can trigger PAP-II expression in DRG neurons. However, the expression pattern of PAP-II induced by inflammation is distinct from that induced by nerve injury. Peripheral inflammation causes PAP-II expression in IB4-positive small DRG neurons, while PAP-II expression can be activated in small DRG neurons shortly after peripheral nerve injury, followed by a shift from small neurons to large neurons during the later post-injury days. These results suggest that PAP-II may play potential roles in the modulation of spinal sensory circuits in pathological pain states.

## Methods

### Animal model and tissue preparation

All interventions and animal care were performed in accordance with the policy of the Society for Neuroscience (USA) on the use of animals in neuroscience research and the guidelines of the Committee for Research and Ethic Issues of International Association for the Study of Pain. The experiments were approved by the Committee of Use of Laboratory Animals and Common facility, Institute of Neuroscience, CAS. All efforts have been made to minimize the number of animals used and their discomfort after peripheral inflammation. Sprague-Dawley male rats (200~250 g, Shanghai Center of Experimental Animals, CAS, Shanghai, China) were anesthetized with sodium pentobarbital (60 mg/kg). Paw inflammation was induced by injection of complete Freund's adjuvant (CFA, Sigma) into the rat plantar subcutaneous space of a hindpaw (200 μl/paw). For peripheral nerve injury model, 5 mm portion of the left sciatic nerve of rats was transected at mid-thigh level. The rats were allowed to survive for 1, 2, 4 and 7 days for inflammation model and 2, 7, 14 and 28 days for nerve injury model (15 rats for each time point for RT-PCR). Axonal transport was studied by ligating the L4 and L5 dorsal roots and proximal portion of sciatic nerve (5 rats) at mid-thigh level 1 day before perfusion fixation. L4 and L5 DRGs of these rats and of normal rats were then dissected and frozen on dry ice. For *in situ *hybridization or immunohistochemistry, the same time course was chosen (6 rats for each time point; 3 rats for *in situ *hybridization and immunohistochemistry respectively), and three normal rats were used as control. The rats were anesthetized and perfused via the ascending aorta with warm (37°C) saline followed by warm solution composed of 4% paraformaldehyde and 0.2% picric acid in 0.1 M phosphate buffer at pH 6.9. The perfusion was then followed by 200 ml of the same fixative (4°C) for another 5 min. L4 and L5 DRGs and the lumbar spinal cord were then dissected out. The tissues were post-fixed in the same fixative for 90 min at 4°C, and were then immersed in 10% sucrose in 0.1 M phosphate buffer for at least 2 days.

### Semi-quantitative RT-PCR

Total RNA of normal rat DRGs and that of inflamed or axotomized rats at different time points were extracted with TRIzol reagent (Life Technologies). The mRNA was purified with an Oligotex mRNA kit (Qiagen). The mRNA (200 ng) of DRGs from normal and inflammatory or axotomized rats was reverse transcribed to cDNA. The PCR products were analyzed on 1.5% agarose gel. Glyceraldehyde-3-phosphate dehydrogenase (GAPDH) was used as control. The primers for detection of mRNA expression were as following: for PAP-I, 5'-TCCTGCCTGATGCTCTTA-3' and 5'-TCATTGTTACTCCACTCCC-3'; for PAP-II, 5'-TGCCCTCTACACGAACCA-3' and 5'-ACTCCACTCCCATCCACC-3'; for GAPDH, 5'-ATCTCCGCCCCTTCCGCT-3' and 5'-TTGAAGTCACAGGAGACAACCT-3'.

### In situ hybridization

A 600 bp digoxigenin-labeled antisense cRNA riboprobe spanning the entire PAP-II or PAP-I coding sequence and 3' UTR was generated from PAP-II cDNA as described previously [52]. DRG sections were fixed in 4% paraformaldehyde for 20 min, treated with proteinase K (10 μg/ml in DEPC water containing 50 mM Tris-HCl, pH 7.5 and 5 mM EDTA) for 20 min, acetylated in 0.25% acetic anhydride/0.1 M triethanolamine (pH 8.0) and prehybridized in hybridization buffer (50% formamide, 5 × SSC, 0.3 mg/ml yeast tRNA, 0.1 mg/ml heparin, 1 × Denhardt's solution, 0.1% Tween-20, 5 mM EDTA in DEPC water) for 4 h at 65°C. The prehybridization buffer was substituted by hybridization buffer with 1 μg/ml of the antisense probe in which the sections were incubated for 14 h at 65°C. After hybridization, excess probe was removed by washing three times with 2 × SSC at 67°C and once with RNase A (1 μg/ml) for 10 min. Sections were then incubated in alkaline phosphatase-conjugated sheep anti-digoxigenin antibodies (1:5,000; Roche Molecular Biochemicals), and then in 1 μl/ml NBT and 3.5 μl/ml BCIP substrates in alkaline phosphatase buffer (100 mM Tris-HCl, pH 9.5, 50 mM MgCl_2_, 100 mM NaCl, 0.1% Tween-20 in distilled water). Control experiments were carried out using a digoxigenin-labelled sense riboprobe for PAP-I or PAP-II.

For quantitative analysis, 2~3 sections (14 μm-thick) from each DRG were representative for each rat and the data were collected from at least three animals at each time point. To determine the percentage of labelled neuron profiles, the number of positive neuron profiles was divided by the total number of neuron profiles. To determine the distribution of labelled neuron within a subset of DRG neurons, the cross-section area from the neuron profiles with a clear nucleus was examined and indexed with 100-μm^2 ^interval. Since the quantitative analysis on the percentage of the labelled neurons was based on profile counts and, therefore, only provides approximate estimates.

### Immunohistochemistry

For all groups, 12 μm-thick sections of the fixed L4 and L5 DRGs, and L4-5 spinal cord segments were cut in series in a cryostat and mounted on same gelatin-coated slides. The sections were processed with indirect immunofluorescence histochemistry. The antibodies were diluted in phosphate-buffered saline with 1% bovine serum albumin and 0.3% Triton X-100. Briefly, the sections were incubated with a mixture of goat anti-PAP-II antibodies (1:200; Lifespan Biosciences) and goat anti-CGRP antibodies (1:5,000; DiaSorin), or rabbit anti-Trk B antibodies (1:5,000; Santa Cruz), or rabbit anti-peripherin (1:5,000; Chemicon) overnight at 4°C. To label IB4-positive small DRG neurons, sections were incubated with fluorescein-labelled IB4 (1:100; Vector Laboratories). After several rinses in PBS, the sections were incubated with fluorescence-conjugated donkey anti-rabbit and rhodamine-conjugated donkey anti-goat IgG (1:100; Jackson ImmunoResearch) for 45 min at 37°C. The sections were rinsed and mounted with a mixture of glycerol/PBS (3:1) containing 0.1% paraphenylenediamine and examined under a Leica SP2 confocal microscope.

For quantitative analysis, 2 sections from each DRG were representative for each rat and the data were collected from at least three animals at each time point. The same calculation for the percentage and the distribution of labelled neuron profiles each DRG was performed as *in situ *hybridization. For axonal transport analysis, the immunofluorescence intensity is measured in the dorsal root and sciatic nerve 500 μm from ligation site.

### Statistical analysis

The data were evaluated by unpaired Student's t-test. All data are shown as mean ± S.E.M. P value < 0.05 was considered to be significant.

## List of abbreviations

PAP: Pancreatitis-associated protein; DRG: dorsal root ganglion; CFA: complete Freund's adjuvant; IB4: isolectin B4; TNF-α: tumor necrosis factor alpha; Reg: regenerating gene; CGRP: calcitonin gene-related peptide; NPY: neuropeptide Y.

## Competing interests

The authors declare that they have no competing interests.

## Authors' contributions

SQH performed immunostaining and *in situ *hybridization of inflammatory animal model with help of QW. JRY and SQH carried out immunostaining of nerve injury animal model. FXZ provided the microarray data. SQH, LB and XZ designed experiments and wrote the manuscript. All authors read and approved the final manuscript.

## References

[B1] Milligan ED Watkins LR Pathological and protective roles of glia in chronic pain Nat Rev Neurosci 2009 10 23 36 10.1038/nrn2533 19096368 PMC2752436

[B2] Marchand F Perretti M McMahon SB Role of the immune system in chronic pain Nat Rev Neurosci 2005 6 521 532 10.1038/nrn1700 15995723

[B3] Hunt SP Mantyh PW The molecular dynamics of pain control Nat Rev Neurosci 2001 2 83 91 10.1038/35053509 11252998

[B4] Kidd BL Urban LA Mechanisms of inflammatory pain Br J Anaesth 2001 87 3 11 10.1093/bja/87.1.3 11460811

[B5] Woolf CJ Costigan M Transcriptional and posttranslational plasticity and the generation of inflammatory pain Proc Natl Acad Sci USA 1999 96 7723 7730 10.1073/pnas.96.14.7723 10393888 PMC33609

[B6] Campbell JN Meyer RA Mechanisms of neuropathic pain Neuron 2006 52 77 92 10.1016/j.neuron.2006.09.021 17015228 PMC1810425

[B7] Hucho T Levine JD Signaling pathways in sensitization: toward a nociceptor cell biology Neuron 2007 55 365 376 10.1016/j.neuron.2007.07.008 17678851

[B8] Xiao HS Huang QH Zhang FX Bao L Lu YJ Guo C Yang L Huang WJ Fu G Xu SH Identification of gene expression profile of dorsal root ganglion in the rat peripheral axotomy model of neuropathic pain Proc Natl Acad Sci USA 2002 99 8360 8365 10.1073/pnas.122231899 12060780 PMC123072

[B9] Iovanna J Orelle B Keim V Dagorn JC Messenger RNA sequence and expression of rat pancreatitis-associated protein, a lectin-related protein overexpressed during acute experimental pancreatitis J Biol Chem 1991 266 24664 24669 1722211

[B10] Okamoto H The Reg gene family and Reg proteins: with special attention to the regeneration of pancreatic beta-cells J Hepatobiliary Pancreat Surg 1999 6 254 262 10.1007/s005340050115 10526060

[B11] Abe M Nata K Akiyama T Shervani NJ Kobayashi S Tomioka-Kumagai T Ito S Takasawa S Okamoto H Identification of a novel Reg family gene, Reg IIIdelta, and mapping of all three types of Reg family gene in a 75 kilobase mouse genomic region Gene 2000 246 111 122 10.1016/S0378-1119(00)00059-7 10767532

[B12] Terazono K Yamamoto H Takasawa S Shiga K Yonemura Y Tochino Y Okamoto H A novel gene activated in regenerating islets J Biol Chem 1988 263 2111 2114 2963000

[B13] Watanabe T Yonemura Y Yonekura H Suzuki Y Miyashita H Sugiyama K Moriizumi S Unno M Tanaka O Kondo H Pancreatic beta-cell replication and amelioration of surgical diabetes by Reg protein Proc Natl Acad Sci USA 1994 91 3589 3592 10.1073/pnas.91.9.3589 8170952 PMC43625

[B14] Zhang YW Ding LS Lai MD Reg gene family and human diseases World J Gastroenterol 2003 9 2635 2641 14669303 10.3748/wjg.v9.i12.2635 PMC4612022

[B15] Narushima Y Unno M Nakagawara K Mori M Miyashita H Suzuki Y Noguchi N Takasawa S Kumagai T Yonekura H Okamoto H Structure, chromosomal localization and expression of mouse genes encoding type III Reg, RegIII alpha, RegIII beta, RegIII gamma Gene 1997 185 159 168 10.1016/S0378-1119(96)00589-6 9055810

[B16] Unno M Yonekura H Nakagawara K Watanabe T Miyashita H Moriizumi S Okamoto H Itoh T Teraoka H Structure, chromosomal localization, and expression of mouse reg genes, reg I and reg II. A novel type of reg gene, reg II, exists in the mouse genome J Biol Chem 1993 268 15974 15982 8340418

[B17] Miyashita H Nakagawara K Mori M Narushima Y Noguchi N Moriizumi S Takasawa S Yonekura H Takeuchi T Okamoto H Human REG family genes are tandemly ordered in a 95-kilobase region of chromosome 2p12 FEBS Lett 1995 377 429 433 10.1016/0014-5793(95)01381-4 8549770

[B18] Sumi S Tamura K Frontiers of pancreas regeneration J Hepatobiliary Pancreat Surg 2000 7 286 294 10.1007/s005340070050 10982628

[B19] Malka D Vasseur S Bodeker H Ortiz EM Dusetti NJ Verrando P Dagorn JC Iovanna JL Tumor necrosis factor alpha triggers antiapoptotic mechanisms in rat pancreatic cells through pancreatitis-associated protein I activation Gastroenterology 2000 119 816 828 10.1053/gast.2000.16491 10982776

[B20] Cavard C Terris B Grimber G Christa L Audard V Radenen-Bussiere B Simon MT Renard CA Buendia MA Perret C Overexpression of regenerating islet-derived 1 alpha and 3 alpha genes in human primary liver tumors with beta-catenin mutations Oncogene 2006 25 599 608 10.1038/sj.onc.1208860 16314847

[B21] Choi B Suh Y Kim WH Christa L Park J Bae CD Downregulation of regenerating islet-derived 3 alpha (REG3A) in primary human gastric adenocarcinomas Exp Mol Med 2007 39 796 804 18160850 10.1038/emm.2007.86

[B22] Dusetti NJ Frigerio JM Fox MF Swallow DM Dagorn JC Iovanna JL Molecular cloning, genomic organization, and chromosomal localization of the human pancreatitis-associated protein (PAP) gene Genomics 1994 19 108 114 10.1006/geno.1994.1019 8188210

[B23] Lasserre C Christa L Simon MT Vernier P Brechot C A novel gene (HIP) activated in human primary liver cancer Cancer Res 1992 52 5089 5095 1325291

[B24] Viterbo D Bluth MH Lin YY Mueller CM Wadgaonkar R Zenilman ME Pancreatitis-associated protein 2 modulates inflammatory responses in macrophages J Immunol 2008 181 1948 1958 18641332 10.4049/jimmunol.181.3.1948

[B25] Vasseur S Folch-Puy E Hlouschek V Garcia S Fiedler F Lerch MM Dagorn JC Closa D Iovanna JL p8 improves pancreatic response to acute pancreatitis by enhancing the expression of the anti-inflammatory protein pancreatitis-associated protein I J Biol Chem 2004 279 7199 7207 10.1074/jbc.M309152200 14660681

[B26] Gironella M Iovanna JL Sans M Gil F Penalva M Closa D Miquel R Pique JM Panes J Anti-inflammatory effects of pancreatitis associated protein in inflammatory bowel disease Gut 2005 54 1244 1253 10.1136/gut.2004.056309 15870231 PMC1774660

[B27] Ortiz EM Dusetti NJ Vasseur S Malka D Bodeker H Dagorn JC Iovanna JL The pancreatitis-associated protein is induced by free radicals in AR4-2J cells and confers cell resistance to apoptosis Gastroenterology 1998 114 808 816 10.1016/S0016-5085(98)70595-5 9516402

[B28] Ogawa H Fukushima K Naito H Funayama Y Unno M Takahashi K Kitayama T Matsuno S Ohtani H Takasawa S Increased expression of HIP/PAP and regenerating gene III in human inflammatory bowel disease and a murine bacterial reconstitution model Inflamm Bowel Dis 2003 9 162 170 10.1097/00054725-200305000-00003 12792221

[B29] de la Monte SM Ozturk M Wands JR Enhanced expression of an exocrine pancreatic protein in Alzheimer's disease and the developing human brain J Clin Invest 1990 86 1004 1013 10.1172/JCI114762 2394826 PMC296822

[B30] Livesey FJ O'Brien JA Li M Smith AG Murphy LJ Hunt SP A Schwann cell mitogen accompanying regeneration of motor neurons Nature 1997 390 614 618 10.1038/37615 9403691

[B31] Nishimune H Vasseur S Wiese S Birling MC Holtmann B Sendtner M Iovanna JL Henderson CE Reg-2 is a motoneuron neurotrophic factor and a signalling intermediate in the CNTF survival pathway Nat Cell Biol 2000 2 906 914 10.1038/35046558 11146655

[B32] Takahara Y Suzuki A Maeda M Kawashima H Nakatani T Kiyama H Expression of pancreatitis associated proteins in urothelium and urinary afferent neurons following cyclophosphamide induced cystitis J Urol 2008 179 1603 1609 10.1016/j.juro.2007.11.034 18295254

[B33] Averill S Davis DR Shortland PJ Priestley JV Hunt SP Dynamic pattern of reg-2 expression in rat sensory neurons after peripheral nerve injury J Neurosci 2002 22 7493 7501 12196572 10.1523/JNEUROSCI.22-17-07493.2002 PMC6757959

[B34] Ampo K Suzuki A Konishi H Kiyama H Induction of pancreatitis-associated protein (PAP) family members in neurons after traumatic brain injury J Neurotrauma 2009 26 1683 1693 10.1089/neu.2008.0847 19351265

[B35] Wallace VC Cottrell DF Brophy PJ Fleetwood-Walker SM Focal lysolecithin-induced demyelination of peripheral afferents results in neuropathic pain behavior that is attenuated by cannabinoids J Neurosci 2003 23 3221 3233 12716929 10.1523/JNEUROSCI.23-08-03221.2003 PMC6742302

[B36] Goldstein ME House SB Gainer H NF-L and peripherin immunoreactivities define distinct classes of rat sensory ganglion cells J Neurosci Res 1991 30 92 104 10.1002/jnr.490300111 1795410

[B37] Amaya F Decosterd I Samad TA Plumpton C Tate S Mannion RJ Costigan M Woolf CJ Diversity of expression of the sensory neuron-specific TTX-resistant voltage-gated sodium ion channels SNS and SNS2 Mol Cell Neurosci 2000 15 331 342 10.1006/mcne.1999.0828 10845770

[B38] Wakisaka S Kajander KC Bennett GJ Effects of peripheral nerve injuries and tissue inflammation on the levels of neuropeptide Y-like immunoreactivity in rat primary afferent neurons Brain Res 1992 598 349 352 10.1016/0006-8993(92)90206-O 1486499

[B39] Landry M Holmberg K Zhang X Hokfelt T Effect of axotomy on expression of NPY, galanin, and NPY Y1 and Y2 receptors in dorsal root ganglia and the superior cervical ganglion studied with double-labeling in situ hybridization and immunohistochemistry Exp Neurol 2000 162 361 384 10.1006/exnr.1999.7329 10739642

[B40] Galeazza MT Garry MG Yost HJ Strait KA Hargreaves KM Seybold VS Plasticity in the synthesis and storage of substance P and calcitonin gene-related peptide in primary afferent neurons during peripheral inflammation Neuroscience 1995 66 443 458 10.1016/0306-4522(94)00545-G 7477885

[B41] Cho HJ Park EH Bae MA Kim JK Expression of mRNAs for preprotachykinin and nerve growth factor receptors in the dorsal root-ganglion following peripheral inflammation Brain Res 1996 716 197 201 10.1016/0006-8993(96)00026-1 8738239

[B42] Lotz M Carson DA Vaughan JH Substance P activation of rheumatoid synoviocytes: neural pathway in pathogenesis of arthritis Science 1987 235 893 895 10.1126/science.2433770 2433770

[B43] Lotz M Vaughan JH Carson DA Effect of neuropeptides on production of inflammatory cytokines by human monocytes Science 1988 241 1218 1221 10.1126/science.2457950 2457950

[B44] Cunha FQ Poole S Lorenzetti BB Ferreira SH The pivotal role of tumour necrosis factor alpha in the development of inflammatory hyperalgesia Br J Pharmacol 1992 107 660 664 1472964 10.1111/j.1476-5381.1992.tb14503.x PMC1907751

[B45] Watkins LR Goehler LE Relton J Brewer MT Maier SF Mechanisms of tumor necrosis factor-alpha (TNF-alpha) hyperalgesia Brain Res 1995 692 244 250 10.1016/0006-8993(95)00715-3 8548310

[B46] Watkins LR Maier SF Goehler LE Immune activation: the role of pro-inflammatory cytokines in inflammation, illness responses and pathological pain states Pain 1995 63 289 302 10.1016/0304-3959(95)00186-7 8719529

[B47] Woolf CJ Allchorne A Safieh-Garabedian B Poole S Cytokines, nerve growth factor and inflammatory hyperalgesia: the contribution of tumour necrosis factor alpha Br J Pharmacol 1997 121 417 424 10.1038/sj.bjp.0701148 9179382 PMC1564704

[B48] Schafers M Geis C Brors D Yaksh TL Sommer C Anterograde transport of tumor necrosis factor-alpha in the intact and injured rat sciatic nerve J Neurosci 2002 22 536 545 11784800 10.1523/JNEUROSCI.22-02-00536.2002 PMC6758659

[B49] Shubayev VI Myers RR Anterograde TNF alpha transport from rat dorsal root ganglion to spinal cord and injured sciatic nerve Neurosci Lett 2002 320 99 101 10.1016/S0304-3940(02)00010-1 11849773

[B50] Sommer C Schmidt C George A Hyperalgesia in experimental neuropathy is dependent on the TNF receptor 1 Exp Neurol 1998 151 138 142 10.1006/exnr.1998.6797 9582261

[B51] Mata M Hao S Fink DJ Gene therapy directed at the neuroimmune component of chronic pain with particular attention to the role of TNF alpha Neurosci Lett 2008 437 209 213 10.1016/j.neulet.2008.03.049 18403116 PMC2668118

[B52] Ding YQ Yin J Kania A Zhao ZQ Johnson RL Chen ZF Lmx1b controls the differentiation and migration of the superficial dorsal horn neurons of the spinal cord Development 2004 131 3693 3703 10.1242/dev.01250 15229182

